# Estimating nutrient uptake requirements for soybean using QUEFTS model in China

**DOI:** 10.1371/journal.pone.0177509

**Published:** 2017-05-12

**Authors:** Fuqiang Yang, Xinpeng Xu, Wei Wang, Jinchuan Ma, Dan Wei, Ping He, Mirasol F. Pampolino, Adrian M. Johnston

**Affiliations:** 1 Ministry of Agriculture Key Laboratory of Plant Nutrition and Fertilizer, Institute of Agricultural Resources and Regional Planning, Chinese Academy of Agricultural Sciences, Beijing, China; 2 Institute of Soil Fertilizer and Environment Resources, Heilongjiang Academy of Agricultural Sciences, Harbin, China; 3 International Plant Nutrition Institute China Program, CAAS–IPNI Joint Lab for Plant Nutrition Innovation Research, Beijing, China; 4 International Plant Nutrition Institute Southeast Asia Program, Penang, Malaysia; 5 International Plant Nutrition Institute, Saskatoon, Canada; Agroecological Institute, CHINA

## Abstract

Estimating balanced nutrient requirements for soybean (*Glycine max* [L.] Merr) in China is essential for identifying optimal fertilizer application regimes to increase soybean yield and nutrient use efficiency. We collected datasets from field experiments in major soybean planting regions of China between 2001 and 2015 to assess the relationship between soybean seed yield and nutrient uptake, and to estimate nitrogen (N), phosphorus (P), and potassium (K) requirements for a target yield of soybean using the quantitative evaluation of the fertility of tropical soils (QUEFTS) model. The QUEFTS model predicted a linear–parabolic–plateau curve for the balanced nutrient uptake with a target yield increased from 3.0 to 6.0 t ha^−1^ and the linear part was continuing until the yield reached about 60–70% of the potential yield. To produce 1000 kg seed of soybean in China, 55.4 kg N, 7.9 kg P, and 20.1 kg K (N:P:K = 7:1:2.5) were required in the above-ground parts, and the corresponding internal efficiencies (IE, kg seed yield per kg nutrient uptake) were 18.1, 126.6, and 49.8 kg seed per kg N, P, and K, respectively. The QUEFTS model also simulated that a balanced N, P, and K removal by seed which were 48.3, 5.9, and 12.2 kg per 1000 kg seed, respectively, accounting for 87.1%, 74.1%, and 60.8% of the total above-ground parts, respectively. These results were conducive to make fertilizer recommendations that improve the seed yield of soybean and avoid excessive or deficient nutrient supplies. Field validation indicated that the QUEFTS model could be used to estimate nutrient requirements which help develop fertilizer recommendations for soybean.

## Introduction

Soybean (*Glycine max* [L.] Merr) is an important dual-purpose crop in China, having a variety of uses as an oil and high-protein crop. Because of its function of biological nitrogen (N) fixation, soybean is also an important crop in rotational cropping systems designed for high yield and efficiency, especially in Northeast China. However, soybean production in China has decreased in recent years because of the lower yield level and lagging technological progress [1]. Fertilizer application has played an important role in increasing yield, but the fertilizer management in current farmers’ practices is not usually in balance with crop demand [2], which limits the soybean yield and results in low nutrient use efficiency [3]. Therefore, a robust fertilizer recommendation method must be established to maximize the soybean yield and improve nutrient use efficiency.

Previous studies on fertilizer recommendations for soybean in China have mainly focused on two categories: soil- and plant-based fertilizer recommendations. Fertilizer recommendation based on soil testing and yield target has been found to increase crop yield and nutrient use efficiency [4]. However, it is costly and time consuming for smallholder farmers to take the numerous soil samples required to capture the substantial heterogeneity of individual fertilization patterns [5]. The plant-based fertilizer recommendation must estimate nutrient uptake to balance crop removal for a specific target yield. Previous studies of crop nutrient estimations usually rely on a single value summarized from few data over large areas, which at times lead to misleading fertilizer recommendations. Furthermore, most of the nutrient management recommendations in the past ignored the interactions of plant nutrients and/or only focused on a single nutrient. However, a detailed nutrient balance is important to develop and sustain modern agricultural systems without incurring human and environmental costs [6].

The quantitative evaluation of the fertility of tropical soils (QUEFTS) model can quantify crop nutrient requirements for a target yield [7]. The model uses a large number of data and takes into full account the interactions between N, phosphorus (P), and potassium (K), so it can avoid the problems arising from the estimations of crop nutrient uptake requirements with limited data [7,8]. And combined with the site-specific nutrient management method, the QUEFTS model uses a linear–parabolic–plateau curve to estimate the relationship between crop yield and plant nutrient uptake in the above-ground dry matter [7,9] and to determine the fertilizer requirements and nutrient management. This combination has been applied to match nutrient supply with crop demand during the growing season in the field [10–13], and the QUEFTS model is a practical tool for the application of site-specific nutrient management [14–17]. It has been successfully implemented to rice [9,18–21], maize [17,22–29], and wheat [16,27,30–32] in many countries, including China, so far. However, estimating the nutrient requirements for soybean with this model has not yet been attempted.

The data in the current literature are an insufficient source of adequate nutrient management information and of fertilizer recommendations for the current level of soybean production in China. Therefore, the purposes of the present study were: (1) to determine the relationships between seed yield and nutrient uptake in soybean across a wide range of yields and environments in China, (2) to estimate the optimal balanced N, P, and K requirements for soybean production in China, and (3) to evaluate the QUEFTS model through fertilizer field experiments of soybean.

## Materials and methods

### Data sources

**Fig 1:**
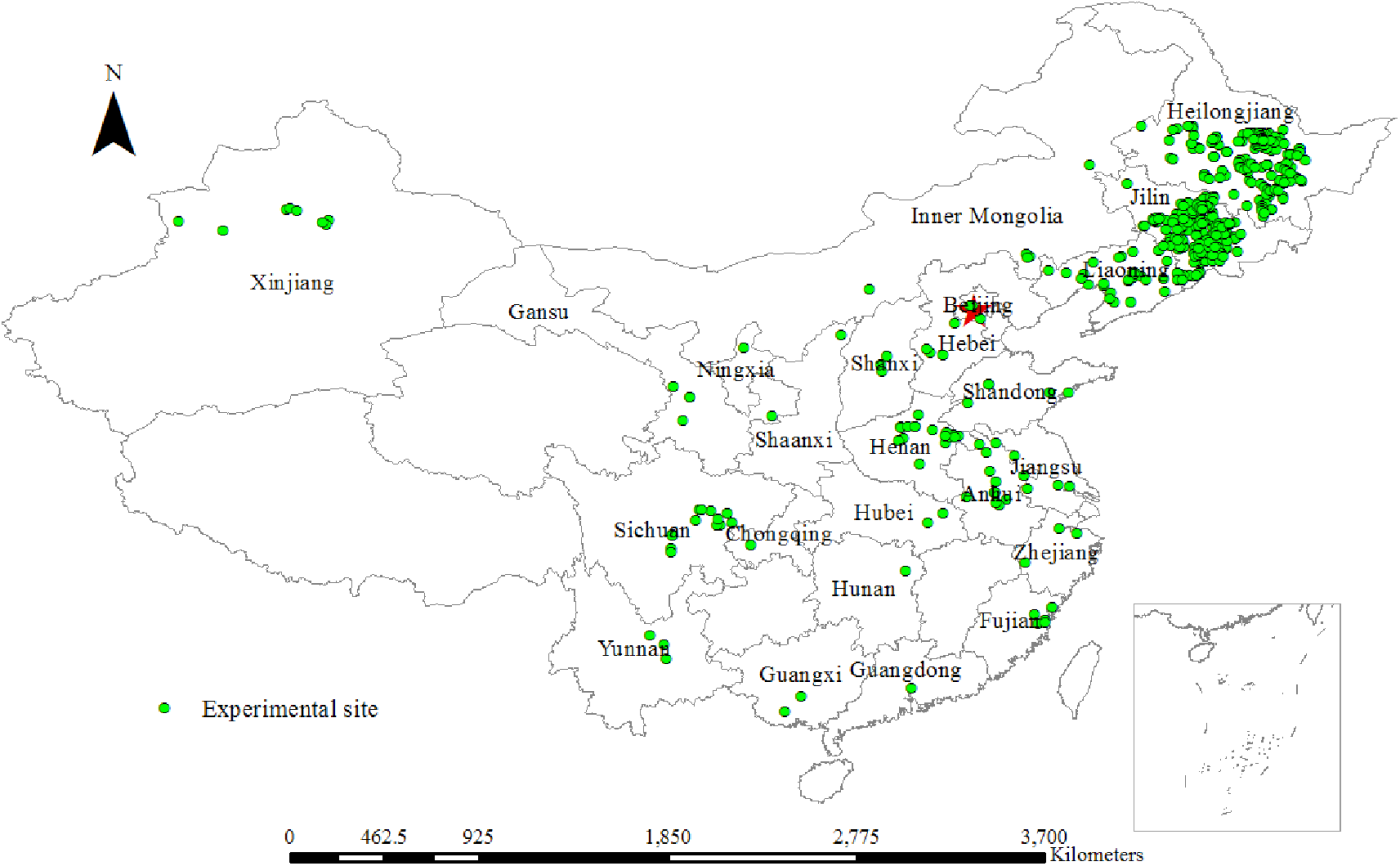
Location map of the experimental sites for soybean (2001–2015) in China.

The database used in this study included field experiments conducted by the International Plant Nutrition Institute (IPNI) China Program, the Program of Modern Agricultural Industry Technology System for Soybean in China and papers published in scientific journals from 2001 to 2015 [33]. The owners of the farmlands gave the permissions to conduct the study on these sites. And the field studies did not involve any endangered or protected species. The experimental sites contained variable nutrient management practices commonly used by Chinese farmers, establishing a wide range of nutrient dilution and accumulation situations, including farmers’ practice, optimal nutrient practices, different fertilizer rates treatments, nutrient omission treatments, and some long-term field experiments across the soybean-growing regions, in 24 Provinces of China (Fig 1). The dataset included the main soil types and climatic conditions for soybean production in China (S1 Table). The soybean varieties in the experiments were all commonly planted in local production and represented the considerable variation that occurred in soybean production areas of China.

### Data analysis

After the data collection and arrangement, we analyzed the seed yield and N, P, and K uptake for all soybean in China, with exclusion of the data with harvest index (HI) value less than 0.4 kg kg^−1^ which were assumed that the crop suffered abiotic or biotic stresses other than nutrient deficiency during the growing season [6], and calculated the maximum nutrient accumulation (*a*) and maximum nutrient dilution (*d*) as the ranges of internal efficiency (IE, seed yield per unit of nutrient uptake in the above-ground parts) based on the actual N, P, and K uptake levels, respectively. These *a* and *d* values were then used as parameters to estimate the nutrient requirements of soybean through the application of QUEFTS model. As described in detail previously [9,16,21,27], the QUEFTS model describes the relationship between crop yield and nutrient requirements [27], and it assumes a constant IE until the yield level increases to 70–80% of potential yield of the crop [34] and the value of IE is used to evaluate the ability of crop to transform nutrients into economic yield [35].

In the present study, we used a solver model based on Microsoft Office Excel that was developed for rice [9], and adapted it to maize [17] and wheat [32]. With the values of *a*, *d*, and target yield input, the model can calculate and produce the optimal balanced N, P, and K requirement of soybean based on the QUEFTS model at different target yields within potential yield levels (3.0–6.0 t ha^−1^). The detail information for the calculation principle of QUEFTS model has been published previously by Janssen et al. in 1990 and 1993 [7,8].

### Field validation

On-farm field experiments were conducted at 20 sites across Northeast China, including Heilongjiang, Jilin, and Liaoning Provinces, in 2014–2015 to validate the QUEFTS model. The region was dominated by a cool temperate climate with a single-cropping system. Soybean was usually sown in late April or early May and harvested in mid- or late September.

The fertilizer recommendations for field validation experiments were provided by the *Nutrient Expert (NE) for Soybean*, a nutrient decision support tool developed by IPNI China Program based on the modified QUEFTS model, and it predicted nutrient uptakes and yield responses to fertilizer applications. The fertilizer N recommendation for soybean was determined by yield response (yield gaps between the plot that received ample N, P, and K and omission plot from which one of the nutrients was omitted) and agronomic efficiency of fertilizer, while the recommendations of P and K rates were determined by the targeted yield and yield response combined with the optimal reciprocal IE (RIE, kg nutrient uptake in above-ground parts per ton of seed) and the nutrient balance required to sustain the soil fertility simulated by the QUEFTS model.

A completely randomized block design with three replications was applied to all experiments. The plot area was 30 m^2^ with the planting density of 12–15 plants m^−2^. Soybean was sown on May 1–10 and harvested on September 20–25 in both test years. Field management was conducted based on the best local management practices and 20–64 kg N ha^−1^, 14–36 kg P ha^−1^, and 23–58 kg K ha^−1^ based on the fertilizer recommendation of *NE* were plowed into the soil before sowing. The sources of N, P, K were urea (46% N), diammonium phosphate (45% P_2_O_5_ and 17% N), and muriate of potash (60% K_2_O). At harvest, plant samples (above-ground parts) with three replicates were harvested to estimate the uptake of N, P, and K for correlation analyses with the QUEFTS model’s simulated uptake, and each plot was manually harvested for measuring the soybean seed yield.

For analysis of the observed nutrient uptake, harvested plant samples, including stems, leaves, shell of the pod, and seed, were oven dried at 80°C for the determination of dry matter weight. Subsamples were digested with H_2_SO_4_-H_2_O_2_ and the N, P, and K concentrations were measured using the Kjeldahl method (B-324, Buchi, Switzerland), vanadomolybdate yellow color method (Cary 100, Varian, Australia), and atomic adsorption spectrophotometer (SpectAA-50/55, Varian, Australia), respectively [36]. The total nutrient uptake levels of N, P, and K were calculated as the products of the nutrient concentration multiplied by the plant dry weight.

The two statistical formulas of root mean square error (RMSE) and normalized-RMSE (n-RMSE) were used to evaluate the QUEFTS model and the deviation between the measured and simulated data. The deviation statistics were defined as follows:

$$RMSE=\sqrt{\frac{\sum_{i=1}^{n}{\left(s_{i}-m_{i}\right)}^{2}}{n}},$$ (1)

$$Normalized\;RMSE=\frac{RMSE}{\bar{m}},$$ (2)

where *s*_*i*_ and *m*_*i*_ are the simulated and measured values, respectively, *n* is the number of data, and $\bar{m}$ is the mean of measured data. The RMSE measures the mean discrepancy between the simulated and measured data with the same unit and the n-RMSE removed the unit and allowed for comparisons between values with different units [37].

SAS software (V8, SAS Institute Inc., USA) was used to further analyze the significance of the difference between the means of simulated and measured values by using least significant difference at 5% probability level.

## Results and discussion

### Yield and nutrient uptake

**Fig 2:**
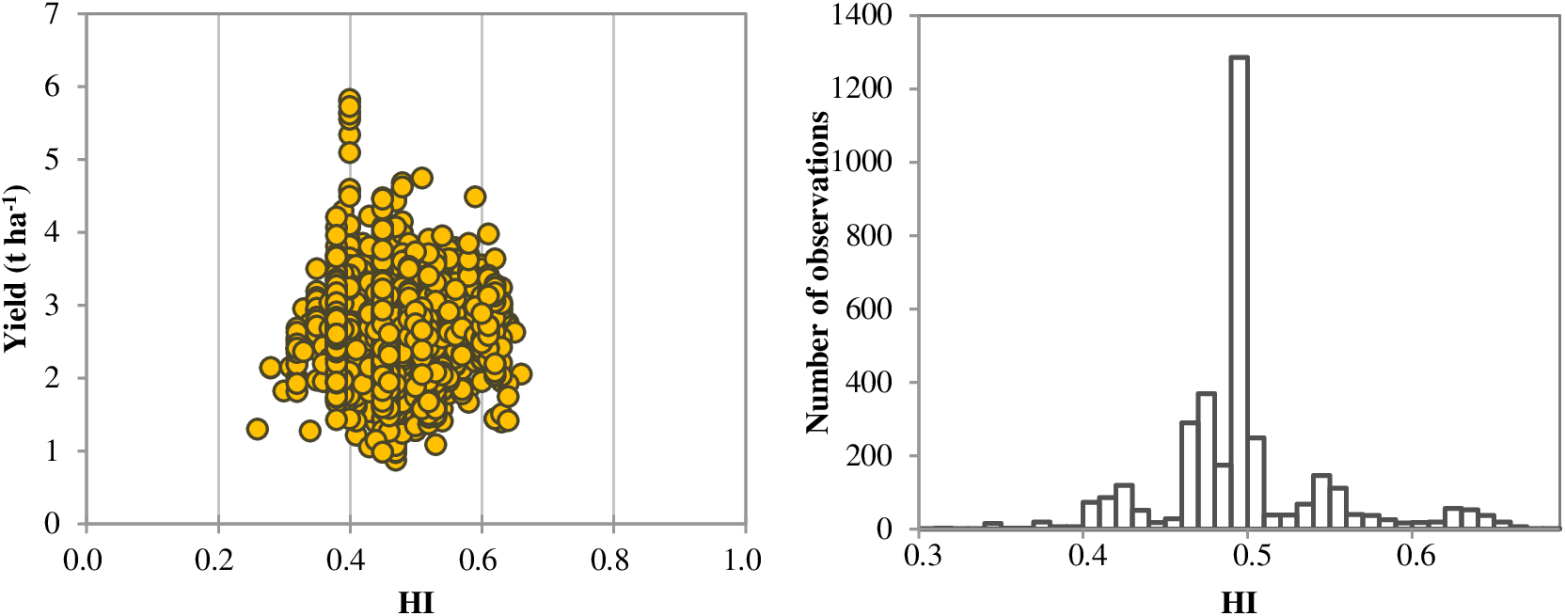
Frequency distribution of the harvest index (HI) of soybean (2001–2015) in China.

The average seed yield (13.5% moisture content) of soybean was 2472 kg ha^−1^ during 2001–2015 in China (Table 1), with a range from 525 to 6514 kg ha^−1^ (including the long-term unfertilized treatment). The average seed yield in the present study was lower than the 2760 and 2700 kg ha^−1^ average yields achieved in the USA and Brazil, respectively, but higher than the 1720 kg ha^−1^, the national average for soybean yield in China from 2001 to 2013 [38] (FAO, 2001–2013). The higher seed yield observed in the present study is from the best field management techniques (including tillage, irrigation, and pest/weeds control, etc.) as compared with normal farmers’ practices. The average of harvest index (HI) was 0.46 kg kg^−1^, with a range from 0.26 to 0.66 kg kg^−1^, and more than 90% of the HI values were between 0.40 and 0.60 kg kg^−1^ (Fig 2).

**Table 1:** Characters of yield and nutrient uptake of soybean (2001–2015) in China.

Parameter	Unit	*n*^a^	Mean	SD^b^	Minimum	25%Q^c^	Median	75%Q	Maximum
Seed yield	kg ha^-1^	9318	2472	683	525	2022	2461	2900	6514
HI	kg kg^-1^	5277	0.46	0.06	0.26	0.42	0.47	0.49	0.66
Shoot N	kg ha^-1^	2193	131.5	38.8	21.1	105.2	125.9	153.1	434.8
Shoot P	kg ha^-1^	2199	21.8	8.6	5.6	16.0	20.3	26.2	72.7
Shoot K	kg ha^-1^	2192	47.6	21.8	8.2	33.5	42.4	56.5	194.4
Seed N	g kg^-1^	2239	53.5	5.7	30.2	50.4	52.0	55.4	122.0
Seed P	g kg^-1^	2239	7.2	2.2	1.7	6.1	7.3	8.2	27.9
Seed K	g kg^-1^	2224	13.3	3.5	2.9	11.0	12.3	14.7	28.2
Straw^d^ N	g kg^-1^	2200	8.9	2.8	1.0	7.0	8.5	10.6	46.4
Straw P	g kg^-1^	2195	3.2	1.9	0.1	2.1	2.9	3.6	15.0
Straw K	g kg^-1^	2187	8.4	3.5	0.6	6.7	8.2	9.5	28.8
HI_N	kg kg^-1^	1570	0.84	0.04	0.71	0.81	0.84	0.86	0.94
HI_P	kg kg^-1^	1579	0.67	0.09	0.42	0.62	0.68	0.71	0.95
HI_K	kg kg^-1^	1576	0.58	0.08	0.36	0.53	0.56	0.61	0.88

^a^ *n* = number of observations

^b^ SD = standard deviation

^c^ Q = quartile

^d^ Straw = stem + leaves + shell of the pod.

The average above-ground nutrient accumulation of N, P, and K were 131.5, 21.8, and 47.6 kg ha^−1^, respectively, and ranged from 21.1 to 434.8, from 5.6 to 72.7, and from 8.2 to 194.4 kg ha^−1^, respectively. The total N and K uptake levels in the present study were far lower than the average values of 219 and 74.4 kg ha^−1^ [39, 40] in the main soybean planting countries, the USA and Brazil, respectively. The lower N and K accumulation levels in soybean found in this study may explain the lower yield in China compared with that in the USA and Brazil.

The average nutrient concentrations in seed were 53.5 g N kg^−1^, 7.2 g P kg^−1^, and 13.3 g K kg^−1^, and those in straw were 8.9 g N kg^−1^, 3.2 g P kg^−1^, and 8.4 g K kg^−1^, respectively. The nutrient concentrations varied substantially in both seed (30.2–122.0 g N kg^−1^, 1.7–27.9 g P kg^−1^, and 2.9–28.2 g K kg^−1^) and straw (1.0–46.4 g N kg^−1^, 0.1–15.0 g P kg^−1^, and 0.6–28.8 g K kg^−1^) because of the different cultivars, environmental conditions, field management practices, and treatments. The variable nutrient concentrations in seed and straw resulted in the highly variable HI of P and K, ranging from 0.42–0.95 kg kg^−1^ and 0.53–0.88 kg kg^−1^, respectively. However, the HI of N was relatively consistent, ranging from 0.71 to 0.94 kg kg^−1^ (Table 1).

### Measurement of the internal nutrient efficiency

The QUEFTS model uses IE and RIE to estimate the relationship between seed yield and nutrient uptake in the above-ground parts of the crop. The measurements of IE and RIE were based on the analysis of several treatments, including optimal fertilizer treatment, omission plots, and farmer’s current practice. The average IEs of N, P, and K were 18.2, 120.3, and 54.2 kg seed yield per kg nutrient uptake for all soybean data, respectively, ranging from 5.2 to 96.0 kg kg^−1^ for N, 39.8 to 441.8 kg kg^−1^ for P, and 20.9 to 217.7 kg kg^−1^ for K (Table 2).

**Table 2:** Internal efficiency (IE, kg seed kg^−1^ nutrient) and reciprocal internal efficiencies (RIE, kg nutrient t^−1^ seed) of N, P, and K of soybean (2001–2015) in China.

Parameter	Unit	*n*^a^	Mean	SD^b^	Minimum	25%Q^c^	Median	75%Q	Maximum
IE_N	kg kg^-1^	2193	18.2	2.6	5.2	17.4	18.4	19.3	96.0
IE_P	kg kg^-1^	2199	120.3	48.0	39.8	95.1	107.4	136.3	441.8
IE_K	kg kg^-1^	2192	54.2	16.5	20.9	46.4	54.2	60.5	217.7
RIE_N	kg t^-1^	2193	55.9	8.7	10.4	51.7	54.4	57.5	194.1
RIE_P	kg t^-1^	2199	9.4	3.2	2.3	7.3	9.3	10.5	25.2
RIE_K	kg t^-1^	2192	19.9	5.8	4.6	16.5	18.4	21.6	47.8

^a^ *n* = number of observations

^b^ SD = standard deviation

^c^ Q = quartile.

To produce 1000 kg of seed yield, the average N, P, and K requirements were 55.9, 9.4, and 19.9 kg in the above-ground parts of soybean, respectively, ranging from 10.4 to 194.1 kg for N, 2.3 to 25.2 kg for P, and 4.6 to 47.8 kg for K. The average values of N and K were lower than those found in previous research studies. Wei et al. (2010) reported that soybean with a higher average yield level (3.0 t ha^−1^) needed 87.6 kg N, 8.3 kg P, and 34.6 kg K to produce 1000 kg seed yield from 1982 to 2002 in China [41]. In addition, Ni (2004) suggested that to produce 1000 kg seed, soybeans required 59.8–70.1 kg N, 14.4–18.1 kg P, and 34.3–44.3 kg K to meet the yield level of 4.5–5.0 t ha^−1^ [42]. The substantial differences in the levels of N and K needed to produce 1000 kg of soybean seed were likely to be related to the yield level; soybean with a higher yield needed greater amounts of N and K to produce 1000 kg seed.

### Parameters for the QUEFTS model

**Fig 3:**
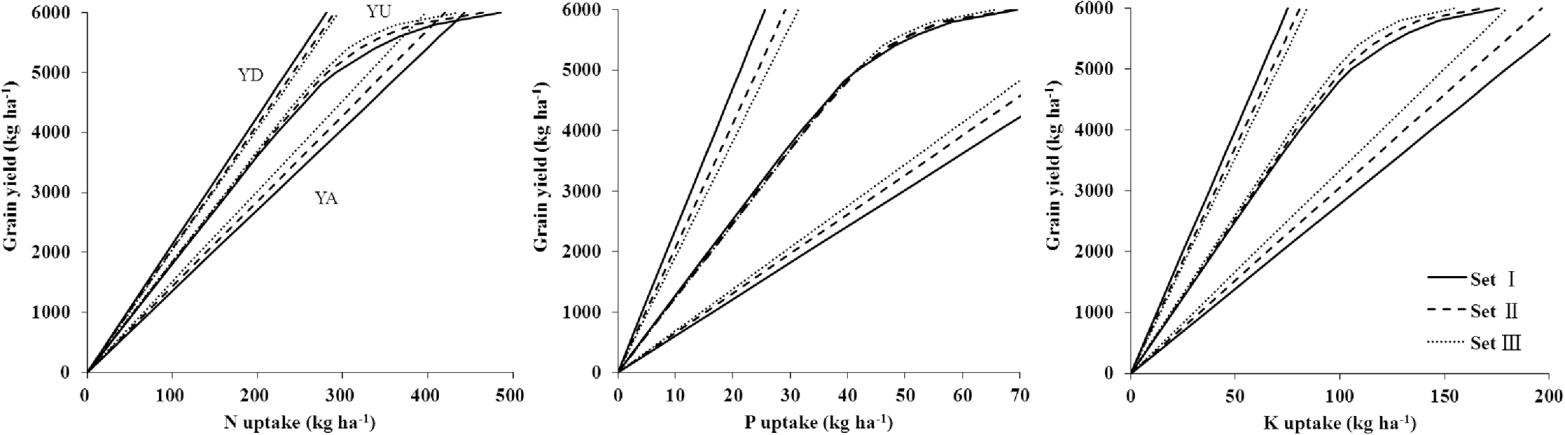
The relationship of seed yield and nutrient uptake of soybean at different sets of constants *a* and *d*. Set I, II, and III excluded the upper and lower 2.5, 5.0, and 7.5 percentiles of all internal efficiency (IE) data, respectively. YD, YA, and YU represented the maximum dilution, maximum accumulation, and balanced uptake of N, P, or K in above-ground parts, respectively. The yield potential was set at 6.0 t ha^-1^ for the present study as an example.

The coefficients *a* and *d* values of N, P, and K were calculated by excluding the upper and lower 2.5 (Set I), 5 (Set II), and 7.5 (Set III) percentiles of nutrient IEs for all soybean data in China (Table 3). The relationship between seed yield and nutrient accumulation in the above-ground parts under a potential yield of 6.0 t ha^−1^ was calibrated with the QUEFTS model to determine the borderlines of *a* and *d* (Fig 3).

**Table 3:** Envelope coefficients of maximum accumulation (*a*) and dilution (*d*) of N, P and K in the above-ground parts for soybean (2001–2015) in China.

Nutrients	Set I		Set II		Set III	
	*a* (2.5th)	*d* (97.5th)	*a* (5th)	*d* (95th)	*a* (7.5th)	*d* (92.5th)
N	13.5	21.4	14.3	20.6	15.1	20.3
P	60.4	234.6	65.4	205.7	68.9	191.0
K	27.8	79.9	30.6	74.1	33.3	71.0

The curves of N, P, and K in the three sets were almost similar (Fig 3), except when the targeted yield approached the yield potential limit. Therefore, Set I was used to estimate the balanced nutrient uptake and the relationship between soybean seed yield and nutrient accumulation in the above-ground parts, because it included a wider range of variability. The constant *a* and *d* values derived from all soybean data in Set I were 13.5 and 21.4 kg kg^−1^ for N, 60.4 and 234.6 kg kg^−1^ for P, and 27.8 and 79.9 kg kg^−1^ for K, respectively. The borderlines of *a* and *d* observed in the present study were different from those reported by Salvagiotti et al. in 2008 (6.4 and 18.8 kg kg^−1^ for N, respectively) [39]. These differences in borderlines of maximum nutrient accumulation and dilution may come from different ecological conditions, soybean cultivars, cropping systems, and management practices, which led to different nutrient uptakes and seed yields. The lower constant *a* and *d* values indicated that more nutrients were needed by crop to produce the same yield [21]. Therefore, the *a* and *d* values in the present study suggest that N is the main limiting factor for soybean yield. This result is consistent with the conclusion of Salvagiotti et al. (2008) [39].

### Estimating the balanced nutrient requirements for soybean

**Fig 4:**
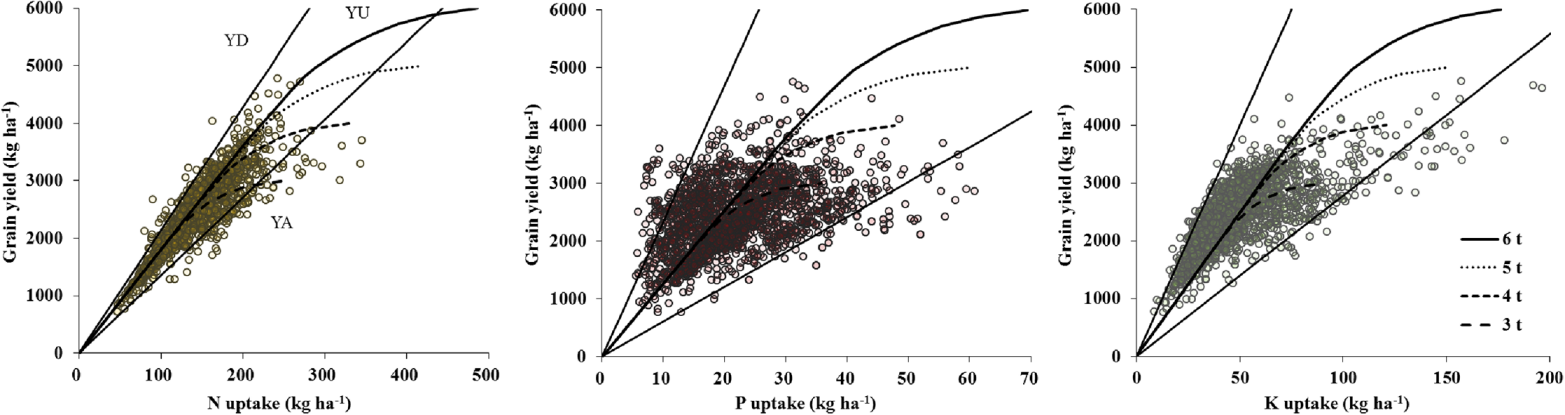
The relationship between seed yield and nutrient accumulation of N, P, and K in above-ground parts at different target yields simulated by the QUEFTS model for soybean in China. YD, YA, and YU represented the maximum dilution, maximum accumulation, and balanced uptake of N, P, or K in above-ground parts for a specific target yield, respectively. The range of yield potential for soybean was from 3.0 to 6.0 t ha^-1^.

The relationship between soybean yield and nutrient accumulation in the above-ground parts at maturity was estimated using the QUEFTS model under different potential yields (Fig 4). The highest yield potential of 6.0 t ha^−1^ was set to run the QUEFTS model to estimate balanced nutrient requirements because the seed yield rarely exceeds this potential yield in China (Fig 2) [43,44]. The model predicted a linear increase in seed yield, until the yield reached about 60–70% of the yield potential, if the different nutrients were taken up in a balanced manner. In another word, whatever the yield potential was, the optimal nutrient accumulation required to produce 1000 kg seed was the same when the yield reached about 60–70% of potential yield (Fig 3).

The QUEFTS model predicted a balanced nutrient accumulation of 55.4 kg N, 7.9 kg P, and 20.1 kg K per ton of seed when the yield reached about 60–70% of the potential yield and an IE of 18.1 kg seed kg^−1^ N, 126.6 kg seed kg^−1^ P, and 49.8 kg seed kg^−1^ K for soybean in China. The optimal N:P:K ratio in the above-ground parts was about 7:1:2.5 (Table 4).

**Table 4:** Nutrient requirements of N, P, and K in total above-ground parts and seed, and removal ratio at different target yields simulated by the QUEFTS model for soybean in China.

Yield	Above-ground requirements			Seed requirements			Ratio in seed		
	N	P	K	N	P	K	N	P	K
t ha^−1^	----------------kg t^−1^----------------						-------------- % ------------		
0	0	0	0	0	0	0	0	0	0
0.8	55.4	7.9	20.1	48.3	5.9	12.2	87.1	74.1	60.8
1.2	55.4	7.9	20.1	48.3	5.9	12.2	87.1	74.1	60.8
1.6	55.4	7.9	20.1	48.3	5.9	12.2	87.1	74.1	60.8
2.0	55.4	7.9	20.1	48.3	5.9	12.2	87.1	74.1	60.8
2.4	55.4	7.9	20.1	48.3	5.9	12.2	87.1	74.1	60.8
2.8	55.4	7.9	20.1	48.3	5.9	12.2	87.1	74.1	60.8
3.2	55.4	7.9	20.1	48.3	5.9	12.2	87.1	74.1	60.8
3.6	55.4	7.9	20.1	48.3	5.9	12.2	87.1	74.1	60.8
3.9	55.7	8.0	20.2	48.3	5.9	12.2	86.7	73.8	60.5
4.2	56.2	8.0	20.4	48.7	5.9	12.3	86.7	73.7	60.5
4.5	56.7	8.1	20.5	49.1	6.0	12.4	86.7	73.7	60.5
4.8	57.3	8.2	20.7	49.6	6.0	12.5	86.6	73.7	60.4
5.0	58.3	8.3	21.1	50.9	6.2	12.9	87.3	74.3	60.9
5.2	60.2	8.6	21.8	52.6	6.4	13.3	87.3	74.3	60.9
5.4	62.5	8.9	22.6	54.6	6.6	13.8	87.3	74.3	60.9
5.6	65.5	9.4	23.7	57.2	6.9	14.5	87.3	74.3	60.9
5.8	70.2	10.0	25.4	61.3	7.4	15.5	87.3	74.2	60.9
6.0	81.0	11.6	29.4	74.6	9.1	18.9	92.1	78.3	64.2

To maintain soil fertility, nutrients removed in the seed or harvested plant parts must be returned to the soil. The calculation of seed nutrient uptake can provide guidance for the appropriate fertilization and avoiding fertilizer waste. Therefore, when making fertilizer recommendations where complete soybean removal is practiced, the removed straw must be considered as well as the seed.

Seed nutrient removal could be also simulated by the QUEFTS model [26]. The model indicated that the balanced N, P, and K removal amounts to produce 1000 kg seed were 48.3 kg N, 5.9 kg P, and 12.2 kg K, respectively, for all soybean data when the targeted yield reached 60–70% of the potential yield, if the seed nutrients were taken up in a balanced manner. Compared to balanced nutrient uptake in total above-ground plant, approximately 87.1%, 74.1% and 60.8% of N, P, and K accumulated in seed and were removed from the field (Table 4). These values should be considered to sustain soil fertility when make a fertilizer recommendation for soybean. In addition, biological N_2_ fixation should also be considered for N fertilizer recommendation, because soybean is relying on biological N_2_ fixation partly. Salvagiotti et al. (2008) thought that 50–60% of soybean N demand was met by biological N_2_ fixation. Therefore, soybean yield with N_2_ fixation was less likely to respond to N fertilizer compared to that without N_2_ fixation [39].

### Field validation

**Fig 5:**
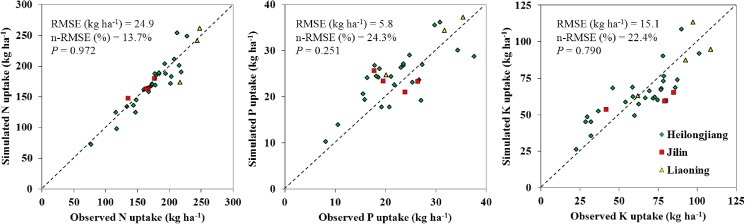
Comparisons of the simulated and observed N, P, and K uptake in soybean. The observed nutrient uptake was from field experiments conducted in Heilongjiang, Jilin, and Liaoning with NE treatments, and the simulated data was from the QUEFTS model.

The relationship between observed and simulated nutrient uptake was analyzed based on the current experiments conducted in 2014 and 2015 in Northeast China. The RMSE and n-RMSE were used to evaluate the quality of fit of the QUEFTS model with the observations. The reference data used for validation were experimental data from actual field trials in which the fertilizer rates were recommended by *NE for Soybean*. The values of RMSE were 24.9, 5.8, and 15.1 kg ha^−1^ for N, P, and K, respectively. The n-RMSE values were 13.7%, 24.3%, and 22.4% for N, P, and K, respectively. While there was some deviation for P and K, the observed and simulated N, P, and K uptake in the above-ground parts occurred near the 1:1 line, and the *P* values for N, P, and K were 0.972, 0.251, and 0.790, respectively, suggesting that the simulated nutrient uptake agreed well with the measured values and that there was no significant difference between observed and model values (Fig 5). The results indicated that the QUEFTS model can be used to predict optimal nutrient uptake which is used to make fertilizer recommendations for soybean.

## Conclusions

The large datasets from a variety range of soybean growing environments was used to estimate the balanced nutrient requirements using the QUEFTS model. The model predicted a linear increase in above-ground parts or seed yield until the yield reached about 60–70% of the yield potential. To produce 1000 kg soybean seed in China, 55.4 kg N, 7.9 kg P, and 20.1 kg K (N:P:K = 7:1:2.5) were required in the above-ground parts, and the corresponding IEs were 18.1, 126.6, and 49.8 kg seed per kg of N, P, and K, respectively. The QUEFTS model also simulated 48.3 kg N, 5.9 kg P, and 12.2 kg K nutrient in seed per 1000 kg seed, accounting for 87.1%, 74.1% and 60.8% of the N, P, and K in total above-ground parts, respectively. The field validation indicated that the QUEFTS model can be used to estimate balanced nutrient requirement which help develop robust fertilizer recommendations for soybean.

## Supporting information

S1 Table Climatic types and soil characters of the experimental sites for soybean (2001–2015) in China. (PDF)

## Abbreviations

HI: harvest index
IE: internal efficiency
K: potassium
N: nitrogen
P: phosphorus
QUEFTS: quantitative evaluation of the fertility of tropical soils
RIE: reciprocal internal efficiency
