# Supplementary material for: Estimating nutrient uptake requirements for soybean using QUEFTS model in China
Source: PLoS One. 2017 May 12;12(5):e0177509. doi: 10.1371/journal.pone.0177509 (PMC5428962; doi:10.1371/journal.pone.0177509)
Supplement: S1 Table — (PDF) [file pone.0177509.s001.pdf]

S1 Table. Climatic types and soil characters of the experimental sites for soybean (2001-2015) in China.

Province	Climatic type	Organic matter (%)	Total N (g kg^{-1})	Olsen P (mg kg^{-1})	Exchangeable K (mg kg^{-1})	Case[a] (*n*)
Heilongjiang	Cool temperate	1.51-78.1	0.11-28.10	4.00-205.00	1.1-515.8	187
Jilin		1.40-63.2	0.16-25.00	0.40-125.20	20.0-274.0	259
Liaoning		0.18-17.82	1.12-2.70	3.10-58.21	67.6-249.0	33
Hebei	Temperate	2.33	1.06	44.50	110.0	5
Shandong		0.93-1.10	0.97-1.85	19.20-81.34	59.5-115.0	7
Henan		0.85-3.78	0.23-1.17	8.10-57.64	68.6-175.7	21
Shanxi		3.43	2.35	7.83	150.0	3
Beijing		3.00	1.02	80.00	150.0	2
Shananxi	Temperate	0.72	6.80	5.10	56.0	1
Inner Mongolia		1.50-1.52	0.60-2.13	30.30-35.00	67.2-140.0	6
Xinjiang		5.38-65.30	0.11-2.15	15.50-124.50	183.0-266.6	9
Ningxia		1.43	0.72	17.42	174.0	1
Gansu		0.82-1.68	0.65-1.33	4.01-7.83	128.0-168.0	5
Anhui	Temperate	1.60-1.77	1.19-1.52	10.10-13.90	104.0-172.0	10
Jiangsu	sub-tropical	3.10-9.58	1.64	18.50-69.00	60.0-110.0	6
Zhejiang		0.96-1.18	0.98	11.50-36.40	95.0-138.0	3
Hubei		1.23	0.76	7.90	122.0	2
Hunan		2.52	1.22	12.00	133.0	1
Sichuan	Sub-tropical	3.93	2.43	27.40	90.0	11
Yunnan		2.48	1.12	18.00	163.0	3
Chongqing		2.43	1.39	2.00	142.0	1
Fujian	Sub-tropical	2.69	0.84	11.10	41.0	5
Guangdong	tropical	1.29	0.69	4.80	50.0	1
Guangxi		3.36	2.20	5.00	79.0	2

[a] The number of samples.
